# Supplementary material for: FERN – a Java framework for stochastic simulation and evaluation of reaction networks
Source: BMC Bioinformatics. 2008 Aug 29;9:356. doi: 10.1186/1471-2105-9-356 (PMC2553347; doi:10.1186/1471-2105-9-356)
Supplement: Additional file 1 — FERN distribution, Version 1.3. This archive contains the FERN source code and binaries as well as documentation and example models in FernML and SBML. [file 1471-2105-9-356-S1.zip › fern/doc/javadoc/fern/simulation/Simulator.html]

Simulator


---


|  |  |  |
| --- | --- | --- |
| **Overview** \| **Package** \| **Class** \| **Use** \| **Tree** \| **Deprecated** \| **Index** \| **Help** | |  |
| PREV CLASS   **NEXT CLASS** | **FRAMES**    **NO FRAMES**     **All Classes** |  |
| SUMMARY: NESTED \| FIELD \| CONSTR \| METHOD | DETAIL: FIELD \| CONSTR \| METHOD |  |


---


## fern.simulation Class Simulator

```
java.lang.Object
  fern.simulation.Simulator
```

**Direct Known Subclasses:**: GibsonBruckSimulator, GillespieEnhanced, GillespieSimple, HybridMaximalTimeStep

---

``` public abstract class Simulator extends Object ```

Base class for stochastic simulators. Extending classes just have to implement  `performStep` which gets invoked as long as the simulation is lasting. They also may
override `initialize` but should in that case call `super.initialize()`
to avoid unwished effects.

Simulators also should handle the special time `theta`: is is a moment in time
when `thetaEvent` is supposed to be invoked (e.g. to measure species populations
at this moment). Consider this, if you want to implement a simulator.

The `fireReaction` are supposed to be invoked when a simulator causes a reaction
to fire.

If an extending class sticks to these conventions, it can take full benefit of the
observer system: One or more `Observer` can be registered at a simulator and
observe certain aspects of the simulation (see the `Observer`s javadoc for more
information).

**Author:**
:   Florian Erhard

---

| **Nested Class Summary** | |
| --- | --- |
| `static class` | `Simulator.FireType`             Defines different types of a firing for reactions. |


| **Field Summary** | |
| --- | --- |
| `protected  double[]` | `a`             Contains the propensities of the reactions. |
| `protected  Stochastics` | `stochastics`             Contains a shortcut to the `Stochastics` framework. |
| `protected  double` | `t`             Contains the actual time of the simulation. |


**Constructor Summary**

`Simulator(Network net)`
  
          Creates a new simulator for the given network.


| **Method Summary** | |
| --- | --- |
| `Observer` | `addObserver(Observer observer)`             Adds an observer to the list of observers. |
| `protected  void` | `fireReaction(int mu, double t_start, double t_end, int times, Simulator.FireType fireType)`             Fires a reaction. |
| `protected  void` | `fireReaction(int mu, double t, Simulator.FireType fireType)`             Fires a reaction. |
| `double` | `getAmount(int species)`             Gets the amount of the given species. |
| `protected  AmountManager` | `getAmountManager()`             Gets the `AmountManager`. |
| `abstract  String` | `getName()`             Gets the name of the algorithm. |
| `Network` | `getNet()`             Gets the simulation network. |
| `double` | `getNextThetaEvent()`             Theta defines a moment, where the simulator has to invoke `theta` of a observer. |
| `double` | `getPropensity(int reaction)`             Gets the current propensity for the given reaction. |
| `PropensityCalculator` | `getPropensityCalculator()`             Gets the `PropensityCalculator`. |
| `double` | `getTime()`             Gets the actual simulation time. |
| `double` | `getVolume()`             Gets the volume of the reaction network. |
| `void` | `initialize()`             Initializes the algorithm: set t=0reset the `AmountManager`recalculate the propensities Gets called at the very beginning of `start` |
| `boolean` | `isInterpolateTheta()`             Gets whether the amount values are returned interpolated for theta events. |
| `abstract  void` | `performStep(SimulationController control)`             Performs one simulation step. |
| `void` | `registerNewTheta(Observer obs, double theta)`             Is called by `Observer.setTheta(double)`. |
| `abstract  void` | `reinitialize()`             Reset propensities when a event has been executed. |
| `void` | `setAmount(int species, long amount)`             Sets the amount of the given species. |
| `void` | `setInterpolateTheta(boolean interpolateTheta)`             Sets whether the amount values are returned interpolated for theta events. |
| `void` | `setVolume(double volume)`             Sets the volume of the reaction network. |
| `void` | `start(double time)`             Starts the simulation up to a given time. |
| `void` | `start(SimulationController control)`             Start the simulation with the given `SimulationController`. |
| `protected  void` | `thetaEvent()`             Gets called when the simulator reaches the special time `theta`. |

**Methods inherited from class java.lang.Object**

`clone, equals, finalize, getClass, hashCode, notify, notifyAll, toString, wait, wait, wait`

**Field Detail**

### t

```
protected double t
```

:   Contains the actual time of the simulation.

---


### a

```
protected double[] a
```

:   Contains the propensities of the reactions.

---


### stochastics

```
protected Stochastics stochastics
```

:   Contains a shortcut to the `Stochastics` framework.


**Constructor Detail**

### Simulator

```
public Simulator(Network net)
```

:   Creates a new simulator for the given network.

    **Parameters:**: `net` - the network to simulate


**Method Detail**

### start

```
public void start(double time)
```

:   Starts the simulation up to a given time. It just uses a `DefaultController`
    and calls `start(SimulationController)`.

    :   **Parameters:**: `time` - simulation time

---


### start

```
public void start(SimulationController control)
```

:   Start the simulation with the given `SimulationController`.

    :   **Parameters:**: `control` - the `SimulationController`

---


### fireReaction

```
protected void fireReaction(int mu,
                            double t,
                            Simulator.FireType fireType)
```

:   Fires a reaction. It calls the observer and the `AmountManager`

    :   **Parameters:**: `mu` - reaction to be fired: `t` - time at which the firing occurs: `fireType` - type of the firing

---


### fireReaction

```
protected void fireReaction(int mu,
                            double t_start,
                            double t_end,
                            int times,
                            Simulator.FireType fireType)
```

:   Fires a reaction. It calls the observer and the `AmountManager`

    :   **Parameters:**: `mu` - reaction to be fired: `t_start` - start of the firings: `t_end` - end of the firings: `times` - number of firings: `fireType` - type of the firings

---


### thetaEvent

```
protected void thetaEvent()
```

:   Gets called when the simulator reaches the special time `theta`. It
    invokes `Observer.theta(double)` of the appropriate observer.

    It has to be called in extending classes.

    It also handles the interpolation.

---


### initialize

```
public void initialize()
```

:   Initializes the algorithm:

    - set t=0
    - reset the `AmountManager`
    - recalculate the propensities

    Gets called at the very beginning of `start`

---


### reinitialize

```
public abstract void reinitialize()
```

:   Reset propensities when a event has been executed.

---


### performStep

```
public abstract void performStep(SimulationController control)
```

:   Performs one simulation step. Between steps the terminating condition is checked.
    The simulators controller is passed, if an extending class wants to check it
    within one step. Implementing algorithms cannot be sure that the propensities
    are correct at the beginning of a step (e.g. if the volume changed). They should
    override `setAmount(int, long)` and `setVolume(double)`
    if they need correct values!

    :   **Parameters:**: `control` - the simulators controller

---


### getName

```
public abstract String getName()
```

:   Gets the name of the algorithm.

    :   **Returns:**: name of the algorithm

---


### getTime

```
public double getTime()
```

:   Gets the actual simulation time.

    :   **Returns:**: actual simulation time

---


### registerNewTheta

```
public void registerNewTheta(Observer obs,
                             double theta)
```

:   Is called by `Observer.setTheta(double)`.

    :   **Parameters:**: `obs` - the observer which is registering: `theta` - the theta to register

---


### getNextThetaEvent

```
public double getNextThetaEvent()
```

:   Theta defines a moment, where the simulator has to invoke `theta` of
    a observer. It is used e.g. to determine the amounts of species at one moments.
    Extending class just have to call `thetaEvent()` which basically
    calls the observer.

    :   **Returns:**: the theta

---


### getAmountManager

```
protected AmountManager getAmountManager()
```

:   Gets the `AmountManager`.

    :   **Returns:**: the `AmountManager`

---


### getAmount

```
public double getAmount(int species)
```

:   Gets the amount of the given species.

    :   **Parameters:**: `species` - species index **Returns:**: amount of species **See Also:**: `AmountManager.getAmount(int)`

---


### setAmount

```
public void setAmount(int species,
                      long amount)
```

:   Sets the amount of the given species. Propensities have to be recalculated!

    :   **Parameters:**: `species` - species index: `amount` - amount of species **See Also:**: `AmountManager.setAmount(int, long)`

---


### getPropensityCalculator

```
public PropensityCalculator getPropensityCalculator()
```

:   Gets the `PropensityCalculator`.

    :   **Returns:**: the `PropensityCalculator`

---


### getNet

```
public Network getNet()
```

:   Gets the simulation network.

    :   **Returns:**: the network

---


### addObserver

```
public Observer addObserver(Observer observer)
```

:   Adds an observer to the list of observers.

    :   **Parameters:**: `observer` - the observer to add **Returns:**: the added observer

---


### getVolume

```
public double getVolume()
```

:   Gets the volume of the reaction network.

    :   **Returns:**: volume

---


### setVolume

```
public void setVolume(double volume)
```

:   Sets the volume of the reaction network.

    :   **Parameters:**: `volume` - the volume

---


### isInterpolateTheta

```
public boolean isInterpolateTheta()
```

:   Gets whether the amount values are returned interpolated for theta events.

    :   **Returns:**: interpolated

---


### setInterpolateTheta

```
public void setInterpolateTheta(boolean interpolateTheta)
```

:   Sets whether the amount values are returned interpolated for theta events.

---


### getPropensity

```
public double getPropensity(int reaction)
```

:   Gets the current propensity for the given reaction.

    :   **Parameters:**: `reaction` - index of the reaction **Returns:**: propensity for the reaction


---


|  |  |  |
| --- | --- | --- |
| **Overview** \| **Package** \| **Class** \| **Use** \| **Tree** \| **Deprecated** \| **Index** \| **Help** | |  |
| PREV CLASS   **NEXT CLASS** | **FRAMES**    **NO FRAMES**     **All Classes** |  |
| SUMMARY: NESTED \| FIELD \| CONSTR \| METHOD | DETAIL: FIELD \| CONSTR \| METHOD |  |


---
